# Factors controlling the reactivity of divalent metal ions towards pheophytin *a*

**DOI:** 10.1007/s00775-017-1472-1

**Published:** 2017-06-21

**Authors:** Ł. Orzeł, J. Waś, A. Kania, A. Susz, D. Rutkowska-Zbik, J. Staroń, M. Witko, G. Stochel, L. Fiedor

**Affiliations:** 1 Faculty of Chemistry, Jagiellonian University, Ingardena 3, 30-060 Kraków, Poland; 2 Faculty of Biochemistry, Biophysics and Biotechnology, Jagiellonian University, Gronostajowa 7, 30-387 Kraków, Poland; 3 Institute of Biology, Pedagogical University of Cracow, Podchorążych 2, 30-084 Kraków, Poland; 4 Jerzy Haber Institute of Catalysis and Surface Chemistry, Polish Academy of Sciences, Niezapominajek 8, 30-239 Kraków, Poland; 5 Institute of Pharmacology, Polish Academy of Sciences, Smętna 12, 31-343 Kraków, Poland

**Keywords:** Metallochlorophylls, Heavy metals, Metal ion insertion, Metal ion activation

## Abstract

In this study, we evaluate the factors which determine the reactivity of divalent metal ions in the spontaneous formation of metallochlorophylls, using experimental and computational approaches. Kinetic studies were carried out using pheophytin *a* in reactions with various divalent metal ions combined with non- or weakly-coordinative counter ions in a series of organic solvents. To obtain detailed insights into the solvent effect, the metalations with the whole set of cations were investigated in three solvents and with Zn^2+^ in seven solvents. The reactions were monitored using electronic absorption spectroscopy and the stopped-flow technique. DFT calculations were employed to shed light on the role of solvent in activating the metal ions towards porphyrinoids. This experimental and computational analysis gives detailed information regarding how the solvent and the counter ion assist/hinder the metalation reaction as activators/inhibitors. The metalation course is dictated to a large extent by the reaction medium, via either the activation or deactivation of the incoming metal ion. The solvent may affect the metalation in several ways, mainly via H-bonding with pyrrolenine nitrogens and the activation/deactivation of the incoming cation. It also seems to affect the activation enthalpy by causing slight conformational changes in the macrocyclic ligand. These new mechanistic insights contribute to a better understanding of the “metal–counterion–solvent” interplay in the metalation of porphyrinoids. In addition, they are highly relevant to the mechanisms of metalation reactions catalyzed by chelatases and explain the differences between the insertion of Mg^2+^ and other divalent cations.

**Electronic supplementary material:**

The online version of this article (doi:10.1007/s00775-017-1472-1) contains supplementary material, which is available to authorized users.

## Introduction

Metalloporphyrinoids play key roles as photocatalytic and catalytic centers, and as carriers of electrons and small molecules in biological systems. Many such metallocomplexes, especially those with transition metal ions, are important catalysts in industry and the laboratory [1], and can be used as photosensitizers in various kinds of phototherapy and diagnostics. Often their functioning relies on those photophysical and photochemical features which result from the interactions of the π-electron system with the central metal ion [2–8]. In addition, the formation of metallosubstituted chlorophylls (Chls) and bacteriochlorophylls (BChls) is relevant from the biological point of view [9] and such modified pigments are useful as model compounds [6, 8, 10]. The chemistry of metalloporphyrins has been extensively explored for over a century and the literature on synthetic approaches to their preparation is indeed vast [11–13].

The impact of metal ion reactivity on metalation of porphyrins has frequently been discussed. Most of these studies, carried out in aqueous solutions, showed analogies between the rates of metalation and water exchange both in simple aquacomplexes [14, 15] and in organic media [16–18], thus pointing to the solvent release step as an important factor in the kinetics of metalloporphyrin formation. Thus, the reaction medium needs to be taken into account, even if it does not explicitly appear in the general reaction equation:

1 $${\text{M}}^{2 + } + {\text{H}}_{2} {\text{P}} = {\text{MP}} + 2{\text{H}}^{ + } ,$$

where M^2+^ is the incoming metal ion, H_2_P is the free base porphyrinoid and M–P is the metalated product. Obviously, the ligands to M^2+^, including solvent molecules, may either increase or lower its activity. For instance, acetate ions act on both partners of the metalation reaction, which underlies the mechanism of the well-known general “acetate method” of porphyrinoid metalation [30].

The synthesis of metalloporphyrinoids in vivo is catalyzed by chelatases, which act to overcome the activation barrier for the insertion of metal ions into free bases [19–22]. The mechanisms of reactions catalyzed by chelatases, i.e. cation recognition, binding and its activation, are not completely understood. In most cases, their catalytic action relies on the activation of the macrocyclic substrate via its deformation in the transition state and clearly, the interior of the enzyme provides a highly controlled reaction medium. Such a deformation seems to occur during metalation in vitro as the activation barrier for metal ion insertion into the free base is correlated with the rigidity of the macrocycle [23]. The reports show opposite trends related to the effect of the size of metal ions on the kinetic and thermodynamic parameters for complex formation [24–26] and two types of macrocycle bending are considered [27, 28]. First, there is the saddle type, in which a bulky metal ion and tetrapyrrolic ligand form the out-of-plane tetradentate complex. Second, the ruffle type is considered, with a “sitting-atop” metal ion coordinated by two core N atoms, while the free electron pairs from the other two are exposed on the opposite side of the macrocycle. The formation of complexes with a bulky metal ion can be expected to facilitate the binding of smaller metal ions [26]. Such a model of “assisted” metal ion insertion shows the essence of the metalation of rigid macrocyclic ligands, such as hemes, Chls and BChls. Recently, we have shown that the reactivity of porphyrinoids in metalation reactions is determined by both the extended aromatic π-electronic system and the peripheral groups, whose interplay creates the energetic barrier to be overcome [29]. Moreover, the type of bonding between the central metal ion and porphyrinoids depends on the structural details of the chelating molecules [6, 8]. Therefore, the effects observed for simple unsubstituted porphyrins cannot be generalized and directly applied to Chls.

In the quest for new metallocomplexes, especially of chemically labile macrocycles and π-electron systems, such as Chls and BChls, and those with less common or inert transition metal ions that would introduce novel and interesting properties, the narrow choice of synthetic methods available becomes limiting. Many reports on the formation of metallo-Chls and metallo-BChls have been published [3, 30–40], but only a few of them provide kinetic and mechanistic details. Often it becomes problematic to compare these results because various approaches and methods that employ different tetrapyrrolic chelators had been used. The knowledge of these aspects is more complete in the case of the metalation of simpler porphyrins, but it is based mostly on reactions carried out in aqueous solutions [23]. As this reaction medium is very different from that offered by the protein interior, they appear only partly related to reactions catalyzed by chelatases in vivo and in vitro and may not apply to water-insoluble porphyrinoids. Therefore, we set out to assess, in a systematic manner, the roles of the solvent and M^2+^, their mutual interactions and macrocycle solvation in controlling the metalation of pheophytin *a* (Pheo*a*). Our approach was to carry out a comprehensive analysis of Pheo*a* metalation using the highest number of M^2+^-solvent combinations possible. Thus, the interactions between Pheo*a* and nine divalent metal ions (Cd^2+^, Co^2+^, Cu^2+^, Hg^2+^, Mn^2+^, Ni^2+^, Pb^2+^, Sn^2+^ and Zn^2+^) in the form of chlorides, acetates and salts with non-coordinating counterions were investigated in several organic solvents. To reveal the environmental effects on the kinetic parameters of metalation, a detailed study on the formation of the Zn–Pheo*a* complex in a series of seven organic solvents was carried out.

The interactions of various divalent metal ions with Chls have recently been investigated computationally but mainly in the context of the transmetalation of the complex [41]. In the present study, because the solvent exchange rate constants relevant to our model system are not available, DFT-based computations were carried out to examine the interactions of the Pheo macrocycle and M^2+^ and estimate the role of solvent in their activation. The present analysis enables us to outline a hierarchy of factors which determine the kinetics of metalation and to identify the mechanisms by which the components of the reaction are activated.

## Experimental

### Materials and methods

#### Pigment preparation

Pheo*a* was prepared from stereochemically pure chlorophyll *a* (Chl*a*), extracted from the cyanobacterium *Arthrospira maxima* and purified following the methods described earlier [29]. Chl*a* was demetalated by a short treatment with glacial acetic acid, which was then evaporated under a stream of N_2_. Pheo*a* was further purified on a short silica gel column using chloroform as the eluent and then stored at −30 °C under argon. All experiments involving pigments were carried out in dim light with freshly prepared solutions.

#### General metalation procedure

The pigment solution (2.5 µM) was mixed in a tandem cuvette with a solution of at least 200-fold excess of metal salt. The reactions were monitored spectrophotometrically until significant changes ceased. In the case of slower reactions, the kinetic traces were derived from the absorption spectra recorded. To minimize the effects of pigment degradation, the time limit for reaction monitoring was set at 24 h. When the progress of metal insertion was negligibly slow compared to that of bleaching, the reaction was deemed not to be occurring. The experiments were carried out at 298 K.

#### Kinetic and spectroscopic measurements

The reactions with metal salts were monitored using Lambda 35 and Lambda 950 spectrophotometers (Perkin Elmer, USA), both equipped with PTP-6 Peltier modules to control the sample temperature. The rapid reactions were monitored on an SX-20 stopped flow system (Applied Photophysics, UK) combined with an LTD6G thermostat (Grant, UK). The electronic absorption spectra were recorded between 350 and 1100 nm.

#### Chemicals

The solvents and metal salts used in the experiments were of analytical or higher grade, purchased from various suppliers: methanol (MeOH), acetonitrile (ACN), *n*-hexane, toluene and 2-propanol from LabScan (Ireland), nitromethane (MeNO_2_), acetone, ethyl acetate (EtAcO), glacial acetic acid, diethyl ether, 1,4-dioxane as well as Cd(NO_3_)_2_, all the chlorides and acetates from POCh (Poland), dimethylformamide (DMF), dimethylsulfoxide (DMSO) and Cu(ClO_4_)_2_ from Sigma-Aldrich (Germany), Zn(CF_3_SO_3_)_2_ and Pb(NO_3_)_2_ from Fluka (Switzerland), Ni(ClO_4_)_2_ and Co(ClO_4_)_2_ from Strem Chemicals (USA). In the kinetic studies, the solvents and the salts were used as supplied.

#### DFT calculations

The density functional theory (DFT) calculations were carried out using the program Turbomole v. 6.3. [42]. The non-local Becke–Perdew functional [43–46] was used to estimate the desolvation energy of Zn^2+^, and a dispersion-corrected B97-D functional [47] to account for the solvation of Pheo*a*. The computations involved geometry optimization of the structures, further confirmed via vibrational analysis. The electronic energies were corrected for zero-point vibrational energy. The Resolution-of-identity (RI) algorithm was used to accelerate computations [48, 49]. All-electron Gaussian type orbitals of def2-TZVP quality were used to define the atomic orbitals [50]. The electronic structures were additionally studied using Lowdin population analysis [51], as in our previous study [29]. The standard convergence criteria for geometry optimization were applied, i.e. 10^−6^ a.u. for the energy, 10^−3^ for the gradient, and 10^−6^ for the root mean square of the density matrix. The computations were carried out at the Academic Computer Centre Cyfronet AGH using PLGrid+ infrastructure.

## Results

The reactions of Pheo*a* with the salts of divalent metal ions were carried out under ambient conditions in MeOH, ACN, MeNO_2_, acetone, EtAcO, DMF and DMSO. This choice of solvents was dictated by both the solubility of the salts and occurrence of the insertion at ambient conditions. The following metal ions, Cd^2+^, Co^2+^, Cu^2+^, Hg^2+^, Mn^2+^, Ni^2+^, Pb^2+^, Sn^2+^ and Zn^2+^, were used in the form of chlorides and acetates, and salts with trifluoromethanesulfonate (triflate, Tf^−^), ClO_4_^−^ or NO_3_^−^, i.e. counterions commonly regarded as non-coordinating. Assuming that in the latter case the solvatocomplexes of M^2+^ are predominant in the reaction medium, the effect of M^2+^ can be separated from the ligand effect. To account for the effect of residual water in the reaction medium, the kinetics of Zn^2+^ insertion was determined in hydrated MeOH containing between 0 and 10% (v/v) water. Apparently, the addition of water to an organic solvent slows down the metalation [Fig. S1 in the Electronic Supplementary Material (ESM)].

**Fig. 1:**
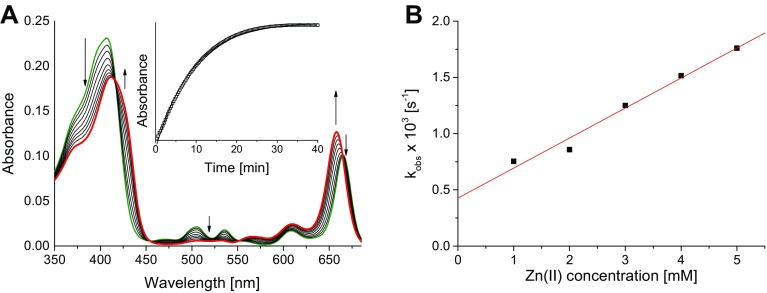
Changes in the absorption spectrum accompanying the reaction of Pheo*a* (*green line*) with Zn(II) chloride in methanol (**a**). *Red line* the spectrum of the final product, Zn–Pheo*a*. Note isosbestic points near 410, 450, 550 and 660 nm. *Inset*: the kinetic trace recorded at 655 nm and 298 K for a 2000-fold excess of Zn(II). Dependence of the *k*_obs_ values on [ZnCl_2_] determined for the reaction at 298 K (**b**)

The reactions were carried out under pseudo-first order conditions secured by the use of a considerable excess of M^2+^. To ensure reasonable reaction rates and enable direct comparison with the results obtained previously [29, 36], the concentration of Pheo*a* was kept constant at 2.5 μM and M^2+^ concentrations ranged between 0.5 and 5.0 mM. The progress of the reactions was followed by monitoring the changes in the Q_Y_ spectral region, as exemplified in Fig. 1. The position of the Q_Y_ maximum is the most indicative as it reflects the occurrence of interactions between the M^2+^ valence electrons and the π-electron system. In most cases, in the course of metalation the Q_Y_ band of Pheo*a* undergoes a hypsochromic shift. In addition, due to the replacement of two core protons in Pheo*a* with M^2+^, the low intensity bands located around 500–550 nm disappear.

Almost all the kinetic traces were satisfactorily fitted with the mono-exponential functions, while in a few cases a double-exponential decay was observed, which assembles the values of *k*_obs_ in the form:

2 $$k_{\text{obs}} = k_{\text{obs}}^{\prime } + k_{\text{obs}}^{\prime \prime } .$$

Such kinetic traces are typical of reactions with redox-active cations, e.g. Cu^2+^, which allow us to associate the slower component with the pigment degradation. The plots of *k*_obs_ and *k*_obs_^′^ against [M^2+^] are linear (see Fig. 1b and Fig. S2 in ESM), and thus a simple rate law:

3 $$k_{\text{obs}} = k_{1} c_{\text{M(II)}} + k_{2} ,$$

can be applied.

### Solvent effect

In a more detailed study, the reactions of Pheo*a* with Zn^2+^, as a representative M^2+^, were analyzed in seven organic solvents, viz. MeOH, ACN, MeNO_2_, acetone, EtAcO, DMF and DMSO. In all cases, ZnTf_2_ was used as the source of Zn^2+^. In MeNO_2_, ACN and acetone the Zn^2+^ insertion runs to completion within minutes and with the second-order rate constants greater than 1 M^−1^ s^−1^ (Table 1). In the other solvents, metalation is markedly slower. The slowest reaction, in DMSO, shows only slight progress after 24 h. Thus, in terms of metalation rates, the following relationships between reaction media were found: MeNO_2_ > ACN > acetone > AcOEt >> MeOH>DMF >> DMSO.

**Table 1:** Kinetic parameters of the reactions of pheophytin *a* and Zn triflate in organic solvents determined from the decay of the Q_Y_ band at 298 K (see the text for details)

Solvent	*τ* ^a^	*k*_obs_ (s^−1^)^a^	*k*_1_ (s^−1^ M^−1^)	*k*_2_ (s^−1^)
MeOH^b^	2.4 h	1.14 × 10^−4^	8.8 ± 1.3 × 10^−3^	7.1 ± 0.4 × 10^−5^
ACN^b^	1.6 min	1.04 × 10^−2^	2.6 ± 0.1	(3.8 ± 0.3 × 10^−1^)^c^
MeNO_2_	5.9 s	1.70 × 10^−1^	3.3 ± 0.2 × 10^1^	6.6 ± 2.8 × 10^−3^
Acetone	2.2 min	7.75 × 10^−3^	1.4 ± 0.1	7.3 ± 1.3 × 10^−4^
Ethyl acetate	11 min	1.49 × 10^−3^	2.9 ± 0.1 × 10^−1^	~0.0
DMF	~18.4 h	1.51 × 10^−5^	1.3 ± 0.1 × 10^−3^	8.9 ± 0.4 × 10^−6^
DMSO	~73.0 h	3.80 × 10^−6^	nd	nd

Values of *k*_1_ and *k*_2_ shown with SD

*nd* not determined

^a^Determined for *c*_Zn(II)_ = 5 mM

^b^Taken from [29]

^c^In (M^−1^ s^−1^)

### Cation and counterion effects

**Fig. 2:**
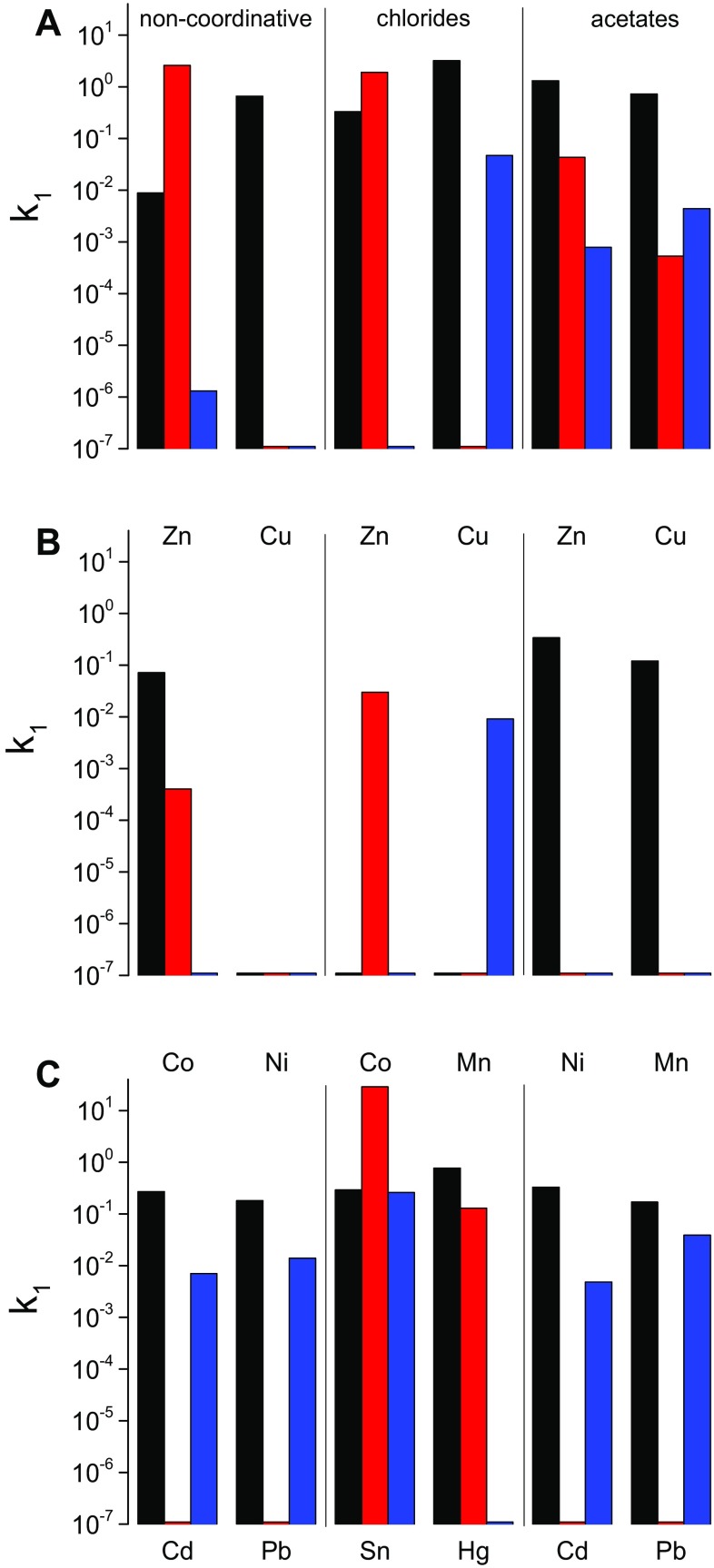
Comparison of Pheo*a* metalation rates for small-sized rapidly incorporating metal ions (**a**), medium-sized slowly incorporating metal ions (**b**) and bulky metal ions (**c**) in methanol (*black bars*), acetonitrile (*red bars*) and dimethylformamide (*blue bars*)

To reveal how the metalation rates depend on the M^2+^ type, reactions of the divalent ions of Co, Cd, Cu, Hg, Mn, Ni, Pb, Sn, and Zn with Pheo*a* were carried out in MeOH, ACN and DMF. To assess the counterion effect, the reactivities of their chlorides, acetates and salts with non-coordinating counterions (each of a similar degree of constitutive hydration) were individually compared. Because Pheo*a* degradation may also affect the kinetics, only the results obtained for reactions completed within 72 h were considered most reliable. In slower reactions, wherever possible, the kinetic parameters were estimated by fitting the tails of the kinetic traces with linear function, and then deducing it from a combined exponentially-linear function used to model the entire trace. The kinetic parameters of the reactions of Pheo*a* with the Cd, Co, Cu, Ni, Pb and Zn salts with non-coordinative counter ions are compared in Fig. 2 and listed in Table S1 (in ESM).

In MeOH, practically all cations undergo insertion and, except Ni^2+^, the *k*_1_ values of their reactions range between 8.8 × 10^−3^ M^−1^ s^−1^ (Zn^2+^) and 6.6 × 10^−1^ M^−1^ s^−1^ (Cu^2+^). The formation of Cd–Pheo*a* and Cu–Pheo*a* is slightly faster than that of Pb–Pheo*a*, and considerably faster than that of Ni–Pheo*a*, which requires more than 150 h to run to completion. The metalations in DMF are generally slower (*k*_1_ values between 1.3 × 10^−6^ and 1.4 × 10^−2^ M^−1^ s^−1^) and Co^2+^ and Ni^2+^ show no sign of insertion after 24 h. Pb^2+^ turns out to insert slightly faster than most of the other M^2+^. The insertion of Cu^2+^ (triflate) is the fastest in the whole series but an excess of it leads to Pheo*a* degradation, due to the high oxidation potential of this cation [52]. The broadest range of metalation rates was observed in ACN, in which hardly any reaction can be observed with Cd^2+^, the formation of Co–Pheo*a* is slower than in MeOH (*k*_1_ = 3.9 × 10^−4^ vs. 7.2 × 10^−2^ s^−1^ M^−1^) and the insertion of Zn^2+^ occurs particularly fast (*k*_1_ = 2.6 × 10^0^ s^−1^ M^−1^).

**Fig. 3:**
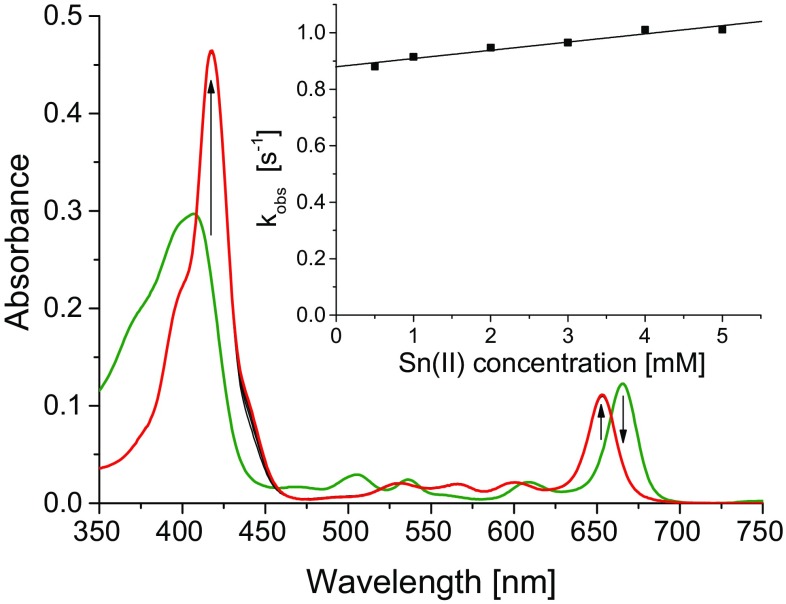
The changes in absorption spectra accompanying the formation of Sn–Pheo*a* (*red line*) from Pheo*a* (*green line*) with SnCl_2_ in acetonitrile. 298 K, 15 min, 2000-fold excess of Sn(II). *Inset*: the dependence of *k*_obs_ on [Sn(II)] determined at 298 K

The interactions of Pheo*a* with the chlorides of Co^2+^, Cu^2+^, Mn^2+^, Sn^2+^, Hg^2+^ and Zn^2+^ were investigated in MeOH, ACN and DMF. In MeOH, Mn^2+^ and Co^2+^ do not react with Pheo*a* at all, while the other cations react quickly, within minutes, with the lifetime of the reaction not exceeding 42 min (Fig. 2 and Table S2 in ESM). In ACN, τ of Co–Pheo*a* formation is 1.8 h, while the reaction with Sn^2+^ is exceptionally fast and occurs within seconds (*τ* ~ 1 s). In the latter case, the reaction could be followed only using the stopped-flow technique (Fig. 3). The significant contribution of the intercept in the observed rate constants (inset in Fig. 3) indicates a competition between two species in reaction with Pheo*a*, probably due to the presence of Sn in two oxidized forms, Sn(II) and Sn(IV) [53]. The insertion of Sn^2+^ is slower in DMF (*τ* = 8.8 min, *k*_1_ = 2.6 × 10^−1^ s^−1^ M^−1^), but still it is faster than that of other cations by at least one order of magnitude.

Due to the poor solubility of the acetates in ACN these salts are not very useful for the synthesis of metallosubstituted Chls in this medium. In MeOH the reactions with acetates are significantly faster and their *k*_1_ values range between 10^−1^ and 10^−2^ s^−1^ M^−1^ (Fig. 2 and Table S3 in ESM). In DMF, compared to MeOH, the insertion rates are much slower and the *k*_1_ values drop by two orders of magnitude, with the remarkable exception of Pb^2+^, whose insertion is the fastest of all cations tested. Cd^2+^ in these two solvents inserts as fast as smaller cations.

### DFT computations of solvent effects in metalation

DFT-based computations served to estimate the energy changes accompanying solvent (MeOH, ACN, acetone and DMF) dissociation from the solvated/coordinated M^2+^, using Zn(solvent)_4_^2+^ as a model complex, in accordance with the equation:

4 $${\text{Zn}}({\text{solvent}})_{4}^{2 + } \rightleftarrows {\text{Zn}}({\text{solvent}})_{3}^{2 + } + {\text{solvent}}.$$

The selection of solvents considered in the computations reflects both the varying rates of metalation (Table 1) as well as differences in their character (protic MeOH, aprotic ACN). As the Lowdin population analysis shows, the net charge on Zn^2+^ in Zn(solvent)_4_^2+^ decreases substantially when changing the ligating/solvating species from ACN to MeOH and then to DMF and acetone, in both the tetra- (ACN 0.44, MeOH 0.31, DMF 0.29 and acetone 0.26) and tri-coordinate (ACN 0.65, MeOH 0.53, DMF 0.48 and acetone 0.46) complexes (Table S5 in ESM). The results of the computations (DFT accuracy ± 2.5 kcal mol^−1^ [54, 55], Table S4 in ESM) indicate that the energetic cost of the first deligation in ACN (44 kcal mol^−1^) is comparable to the removal of DMF (42 kcal mol^−1^), while the acetone and MeOH molecules are easier to dissociate (each 39 kcal mol^−1^). The complete desolvation of Zn(MeOH)_4_^2+^, which yields free Zn^2+^ and 4 MeOH molecules, is the easiest in the set studied (314 kcal M^−1^). The desolvation of Zn(acetone)_4_^2+^ and Zn(ACN)_4_^2+^ is energetically more demanding (361 and 375 kcal M^−1^, respectively), while the complex with DMF is the most stable (399 kcal M^−1^).

**Fig. 4:**
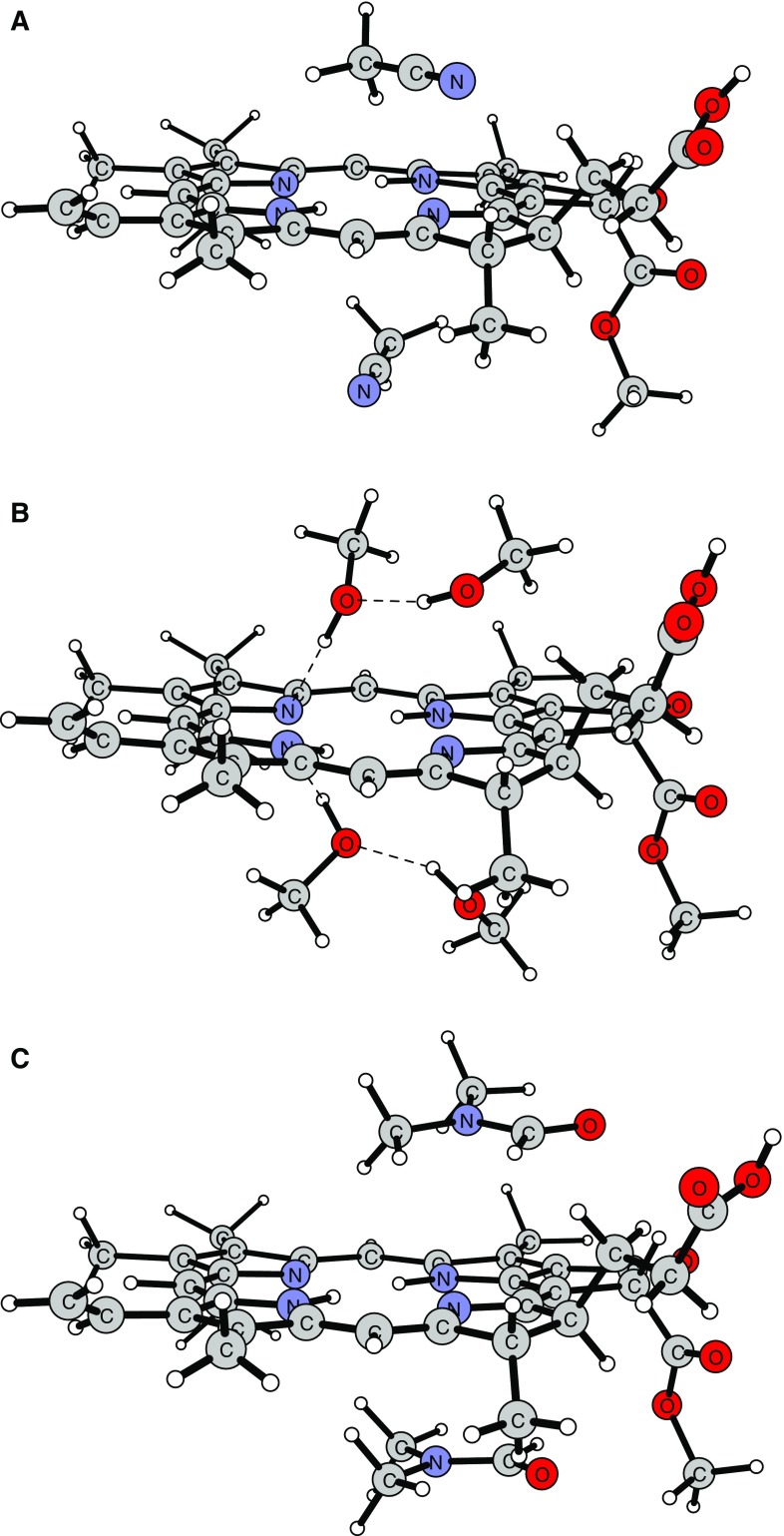
The DFT optimized geometry of pheophytin *a* in acetonitrile (**a**), methanol (**b**), and dimethylformamide (**c**). Note the network of hydrogen bonds formed by methanol around the central cavity and the interaction with the dimethylformamide molecule (see the text for details)

Next, the solvation of the Pheo*a* system was considered. In the calculations, the number of solvent molecules was increased gradually from two to twelve, but qualitatively the results did not change above two molecules. Additional solvent molecules were either placed further from the center of the macrocycle or formed a second solvation sphere. Therefore, for simplicity, only the systems containing Pheo*a* and two solvent molecules (ACN or DMF) and four (for MeOH, due to the strong H-bonding interactions between solvent molecules) are presented. Their geometries were optimized with the use of the DFT method applying a dispersion-corrected B97-D functional to account for the weak solute–solvent interactions. The resulting structures are shown in Fig. 4. The solvent-induced changes in the angles between the “meso” carbon atoms are rather minor (2.1° in ACN, 2.0° in MeOH, and 1.8° in DMF), while larger differences are observed in the torsion angles between the N–H bonds and the macrocycle plane. The largest deformation (13.3°) is found in MeOH, which is the strongest H-bond donor. The effects of other solvents are weaker, amounting to 4.5° in ACN and 3.0° in DMF. The DFT calculations also predict that MeOH molecules do form H-bonds with the pyrrolenine Ns and may block the central cavity (see Fig. 4), which concurs with experimental observations [56].

## Discussion

### Experimental model system

Addressing the question of how the M^2+^ properties determine metalation rates requires a comparison of kinetic data from a series of reactions in which M^2+^ is the only variable. This, in practical terms, is quite limiting, because the M^2+^ oxidation state, the solvent and the counter ion need to be invariant or at least very similar along the series. Most of these requirements are met in the present model system comprising the metalation of Pheo*a* in organic solvents, which facilitates the comparative kinetic analysis of a large set of reactions.

The metalation rate constants were determined under pseudo-first order conditions as they provide a direct comparison of the effects of M^2+^. At large excess of M^2+^, the kinetics of Pheo*a* metalation in all systems tested can be satisfactorily described by a simple Eq. 3. In the most complex case, i.e. Cu^2+^, it has a double set of parameters (Eq. 2), which indicates the contribution of at least two processes to the overall kinetics. The minor one is either a slow macrocycle degradation [52] or it is a parallel formation of Cu^2+^–Pheo*a*, e.g. by some less-populated metal species present in a particular solvent [36, 52]. The slower components, contributing to $k_{\text{obs}}^{\prime \prime }$, may be disregarded in the subsequent discussion, because $k_{\text{obs}}^{\prime } \gg k_{\text{obs}}^{\prime \prime }$. All the other systems can be satisfactorily approximated to the simple rate law (Eq. 3), in which the *k*_2_ term can be assigned to a parallel rather than a reverse reaction due to the following: (1) the kinetics profiles are independent of [M^2+^], (2) M^2+^–Pheo*a* complexes in non-acidic solutions are stable [29, 36, 57, 58], and (3) the metal ions used have complex speciation in organic solvents [36, 52]. The [M^2+^]-independent reaction most likely relates to metalation involving minor coordinative forms of M^2+^.

### Solvent effect

**Fig. 5:**
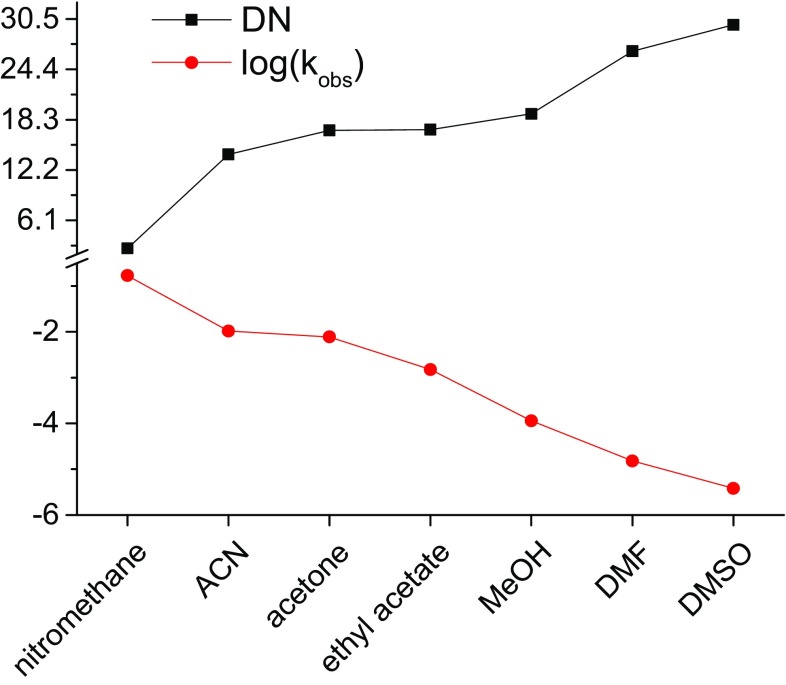
The comparison of the rates (as log(*k*_obs_) for [M^2+^] = 5 mM at 298 K) of Zn–Pheo*a* formation from Pheo*a* and Zn triflate in a series of organic solvents with the values of Gutmann’s donor number (DN)

The kinetics of Zn–Pheo*a* formation in a series of solvents shows that the more innocent the medium (i.e. less coordinative), the faster M^2+^ incorporation, which seems roughly to correlate with both the increasing solvent nucleophilicity and a reverse proportionality of the logs of *k*_obs_ to Gutmann’s donor numbers (Fig. 5). This indicates that the activation energy of the rate-limiting step, i.e. the formation of the first M^2+^–N bond, can be widely modulated by solvation. In terms of thermodynamics, the solvent affects Δ*G* ^≠^ of metalation in two ways: (1) solvation of the reactants contributes to Δ*S* ^≠^ and (2) solvent–macrocycle interactions facilitate conformational changes which affect Δ*H* ^≠^. A strong metal ligation, i.e. an increase in the coordination number in the activated complex, lowers Δ*S* ^≠^. A similar but weaker effect on the entropic component is expected to come from strong interactions in the solvation spheres of the reactants. At the same time, depending on whether they block the central pocket or expose the donor atoms of the macrocycle, specific solvent–macrocycle interactions will either lower or increase Δ*H* ^≠^.

The second-order rate constant (*k*_1_) for Zn^2+^ insertion in DMF is 3 and 6 orders of magnitude lower than in MeOH and ACN, respectively. An even greater difference can be seen in the rate constants for metalation with other M^2+^ (Tables S1–S3 in ESM). Indeed, the electrostatic interactions are weaker in DMF (*ε* ~ 37; Table S5 in ESM) than in MeOH and ACN (*ε* ~ 33), which should lower the driving force for metalation. The low reactivity of Zn^2+^, at least in DMF, can be rationalized by the DFT-based predictions, as DMF molecules are the most difficult to dissociate from Zn^2+^. In general, the desolvation energies correlate to the kinetic parameters of the reactions with Zn^2+^ in the solvents studied, in a similar way to that reported previously [59, 60]. However, the theoretical predictions do not entirely agree with the experimental observations, which suggests that in addition to solvation of M^2+^ there are some other factors at play, such as the formation of an electrostatically stabilized complex between the solvated metal ion and the tetrapyrrolic macrocycle, known as the sitting-atop complex (SAT) [30, 59, 61]. The Lowdin population analysis shows (Table S5 in ESM) that the experimental trend in ZnTf_2_ reactivity (ACN > MeOH > DMF) reflects the modulation of the positive charge on Zn^2+^ by its solvation sphere, which corresponds to a reciprocal relationship between solvent donor number and M^2+^ reactivity (Fig. 5).

The solvent acceptor number seems to play a minor role. These results are in line with previous studies which showed that the solvent is a key factor controlling the rates of metal exchange in porphyrinoids, mostly via the solvation of metal ions [30, 36, 61]. Yet, specific solvent–macrocyclic ligand interactions also play a role [29], and because the degree of macrocycle distortion is directly related to the value of Δ*H* ^≠^ of metalation [18, 29, 62], one should consider the geometrical factor in solvent–porphyrinoid systems. Such effects are seen in the acetate-assisted transmetalation of Chl*a* [30] and have been indicated in computational studies on Chl*a* ligation [63], ascribed to solvents that can stabilize the pentadentate structure of Chl*a*. Also H-bonding to the core Ns may result in a slight distortion of the macrocycle or, at least, out-of-plane deviation of the electron pairs on the core Ns, which is expected to increase the M^2+^–tetrapyrrole interface [36]. On the other hand, because the incorporation of metal ion into free base porphyrin involves a rate-determining solvent exchange with the core Ns, followed by faster proton substitution [17, 36, 61], those protic solvents which are likely to interact with the core Ns will be stronger inhibitors of metalation than the H-bond acceptors. Thus, the low values of the rate constants of the reaction in MeOH can in part be attributed to this type of solvent-induced deactivation of the macrocycle. The present computational analysis shows the formation of H-bonds between MeOH and the chelator (Fig. 4), and predicts that solvent-induced ring deformation will correlate with the strength of H-bonds. The largest deformation occurs in MeOH, which was also noted experimentally [56]. Similarly, H-bonding to pyrrolenine Ns and hindrance of the central cavity due to DMF molecules leads to lower reaction rates in this solvent as compared to ACN. The interactions between the macrocycle and DMF, obviously a bulkier and electron-rich molecule, are expected to be strong, while ACN, not able to H-bond, leaves the core Ns prone to the attack of incoming metal ion. H-bonding may be another reason for the significant difference in the *k*_1_ values for Zn^2+^ insertions in MeOH and ACN (Table 1). Yet, the kinetics of metalation in MeOH is not entirely consistent with the one determined in ACN (Tables S1–S3 in ESM), which, together with considerable differences between metal ions in the reaction rate constants, point to the dominant role of M^2+^–solvent over M^2+^–macrocycle interactions. The effect of H-bonding is less straightforward when one considers the reactions with other cations. In these cases, some other factors seem to be critical, e.g. the valence configuration of the ion, its ionic radii, charge density or the coordination strength of the counterions (see below). Further complications occur in solvents that favor complex speciation of M^2+^.

### Incoming metal ion

The use of salts with non-coordinating counterions provides direct information about the impact of M^2+^ properties on metalation rates (Tables S1–S3), which kinetically order the cations as follows: Cu^2+^ ~ Zn^2+^ > Cd^2+^ > Ni^2+^ > Pb^2+^ ~ Mn^2+^, and allow them to be classified into several groups (Fig. 2). Both Cu^2+^ and Zn^2+^ insert exceptionally rapidly, regardless of solvent and salt type. Their very high reactivity may be related in part to their ionic radii, 0.65 Å for Cu^2+^ and 0.68 Å for Zn^2+^ [64], matching that of the inner cavity. However, the insertion of the latter is somewhat slower and slows down in the presence of weakly coordinative counterions. This shows that the dependence of the reaction rate on M^2+^ may be underestimated for chlorides and in particular for acetates (see below), in line with predictions based on the Irving–Williams series [15, 16, 65].

The second class, including Ni^2+^, Co^2+^ and Mn^2+^, shows moderate rates of insertion and, typically, the second-order rate constants in this group vary between 10^−4^ and 10^−7^ M^−1^ s^−1^. This makes kinetic analysis difficult as metalation may compete with chelator degradation. The differences in the metalation rates between Zn^2+^ and Ni^2+^/Co^2+^/Mn^2+^ could be due in part to the fact that the latter cations form a mixed, i.e. coordinative-covalent type of bonds with the core Ns in Pheo*a*, in contrast to Zn^2+^, bound via purely coordinative bonds [6]. The insertion of these cations requires their valence configuration to be rebuilt along the reaction pathway, which may elevate the energetic barrier for this reaction.

The ions of heavy metals—Hg^2+^, Pb^2+^ and to a lesser extent Cd^2+^—belong to another, more specific group. They do bind to Pheo*a* with second-order rate constants of either near 10^−1^ M^−1^ s^−1^ or well below 10^−7^ M^−1^ s^−1^. Because of their large ionic radii, 0.96, 0.98 and 0.78 Å, respectively [64], they hardly form “in plane” complexes, such that their binding is usually accompanied by a considerable macrocycle distortion. The rates of M^2+^–Pheo*a* formation with these cations were found to be considerably higher than those of smaller ionic radii (Tables S1–S3 in ESM), which again indicates the key role of macrocycle distortion in metalation [17, 25, 26, 66]. Also the lower metalation rates seen for Pheo*a*, as compared to porphyrins, may reflect an increased rigidity of its macrocycle. A separate issue is the unusually fast rate of the reaction between Pheo*a* and SnCl_2_ in ACN. The large ionic radius of Sn^2+^ (0.93 Å in the 6-coordinate form [67]) favors the formation of non-planar complexes with tetrapyrroles, especially taking into account the ability of Sn^2+^ to form tetrahedral and pyramidal complexes. Furthermore, SnCl_2_ is one of the most potent Lewis acids [64, 68].

### The counterion

One of the major concerns when choosing a counterion suitable for the synthesis of metallosubstituted (B)Chls is its capacity to lower the oxidation potential of M^2+^. A comparison of reactions with three types of counterion, non-coordinating (perchlorate, triflate and nitrate), coordinating monodentate (Cl^−^) and chelating (AcO^−^) reveals drastic differences. For instance, Ni^2+^ with non-coordinative counterion in DMF shows no reaction at all while NiCl_2_ reacts very fast. In other cases, the reaction rates differ by several orders of magnitude, depending on the counterion (Fig. 2 and compare data in Tables S1, S2 and S4 in the ESM). Interestingly, the solvatocomplexes of Zn^2+^, Cu^2+^ and Co^2+^ react with Pheo*a* in MeOH considerably more slowly than their chlorides and acetates, both prone to form less reactive complexes with M^2+^ due to their charge. Acetate, even if it accelerates transmetalation [30], in solution forms inert binuclear (or polymeric) complexes that lower the effective concentration of reactive M^2+^, but importantly, it prevents macrocycle oxidation. This makes the acetate/MeOH system an efficient and versatile medium for the preparation of metallochlorophylls [30, 36].

The explanation of the counterion effect is not straightforward for the following reasons: (1) the ligand exchange rates are faster than the formation of the first M^2+^–Pheo*a* bond, (2) a relatively low population of inert [M_2_(AcO)_*n*_]^4−*n*^ complexes, (3) the presence of some vacant coordination sites on mononuclear Cl^−^/AcO^−^ complexes, and (4) the solvation of counterions. The latter effect could contribute to the higher rates of the reactions with Cu(AcO)_2_ and Zn(AcO)_2_ in MeOH than in ACN.

The fate of counterion in reaction medium may also be of some importance when a large excess of salt is applied. Chloride and triflate in polar solvents are likely to remain in the ionic form, whereas AcO^−^, a strong nucleophile, will tend to capture protons from the environment. In aprotic solvents, the free base Pheo*a* may be the source of protons, which will promote the progress of the reaction by affecting the equilibrium described in Eq. 1. For the same reason, i.e. due to its high basicity [69], MeOH can be considered the most favorable of the solvents used.

**Fig. 6:**
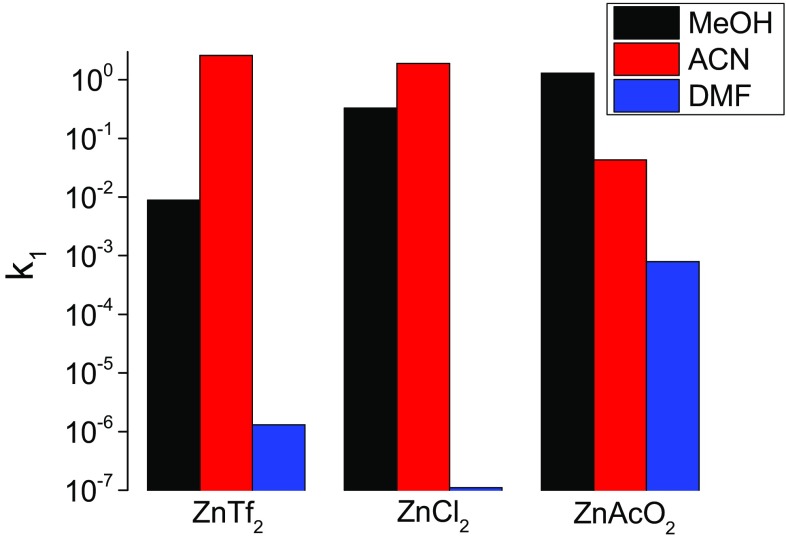
Comparison of the second-order rate constants of Pheo*a* metalation with zinc triflate (Tf), chloride and acetate (AcO) in methanol (MeOH), acetonitrile (ACN) and dimethylformamide (DMF)

The comparison of the kinetics of Zn–Pheo*a* formation using three salts of Zn^2+^, triflate, chloride and acetate, in MeOH, ACN and DMF, as presented in Fig. 6, provides additional information. Clearly, the reactivity of Zn^2+^ is a product of many factors and the effects of counterions seem to depend on solvent properties. In MeOH, which is both a relatively good ligand as well as H bond donor, the reactivity of Zn salts increases in the following way: Tf^−^ < Cl^−^ < AcO^−^. This can be explained by the destabilization of the Zn–AcO mononuclear and inert polymeric complexes due to solvation. A reverse trend is observed in ACN, a poorly coordinating and solvating medium, in which the metalation rate is inversely proportional to the coordinative properties of the counterion. Such dependencies are less obvious in DMF, but the exceptionally low rate constant of Zn–Pheo*a* formation with ZnCl_2_ may be associated with complicated speciation, which, by analogy to CuCl_2_ [65], significantly reduces the concentration of Zn^2+^ reactive species.

### Mg^2+^ insertion in biological systems

Any direct comparison between the insertion of Mg^2+^ and the metal ions discussed above is not really feasible due to the fact that the former does not spontaneously insert into Pheo. Indeed, the insertion of Mg^2+^ into protoporphyrin IX requires extraordinary components and high amounts of energy [70, 71]. This energy is used to transfer Mg^2+^ from its output complexes, most likely aquacomplexes, to the enzyme’s binding site, presumably formed by two serines, aspartic acid and threonine [72]. Such conditions cannot be achieved in a simple in vitro system easily. The high energy demand of this process seems to be related to the fact that Mg^2+^ is bound in the central cavity of porphyrinoids not by coordinative bonds but by much weaker electrostatic forces [6]. As a consequence, in the course of Mg^2+^ insertion a significant change in its coordination state must take place, from a (most likely) hexacoordinated species to a singly or doubly axially coordinated ion electrostatically bound in the central cavity. Large amounts of energy in the form of ATP would be required first to activate the Mg^2+^ ion by stripping off its coordination sphere and then to protect this reactive species during its attack on the enzyme-bound protoporphyrin IX.

## Conclusions

By applying the same approach across many solvents and ions in the model system for pheophytin *a* metalation, we were able to reveal and compare the key factors that influence the kinetics of this reaction. Both the solvent, as the reaction medium, and the counterion to the incoming metal cation, have to be regarded as decisive participants in the reaction, able either to inhibit or greatly facilitate the metalation of porphyrinoids. Hence, the overall reaction is certainly an interplay of various factors, with the energetic, steric and electrostatic effects coming from all participants in the process. The M^2+^ size brings about the clearest effect on the metalation rate, which may dominate over other factors. In this respect, the series of metal ions investigated can be divided into three classes: (1) small-sized cations which incorporate rapidly (Zn^2+^ and Cu^2+^), (2) medium-sized cations which incorporate rather slowly (Ni^2+^, Co^2+^ and Mn^2+^), and (3) bulky metal ions which insert rapidly; these latter form non-planar complexes (Cd^2+^, Hg^2+^ and Pb^2+^).

On the solvent side, several factors have to be considered. Metalation is retarded by any strong interaction of incoming M^2+^ with solvent molecules as well as solvent H-bonding to pyrrolenine nitrogens that increase the steric hindrance for an incoming M^2+^ and contribute to higher activation energies of Pheo*a* metalation. On the other hand, the increased exposure of pyrrolenine nitrogens, due to the interactions with solvent molecules, may render Pheo*a* prone to M^2+^ attack. In addition, the rate of formation of the first M^2+^–N bond is controlled by solvation.

The comparison of the metalation rates shown in Fig. 2 leads to other general conclusions. The effects of counterion and solvent seem to be diminished with bulky metal ions. Apparently, the large ionic size favors the associative mechanism of ligand substitution and reduces the importance of the leaving group. Moreover, large M^2+^ in the out-of-plane position may retain some of their original ligands, in contrast to relatively small ions that do not exceed the size of Mg^2+^. The differences in solvent donor properties, reflected in both the coordination and solvation, must play a more significant role, at least because of the need to release specific binding sites for pyrrolenine nitrogens.

The present results have relevance to the synthetic applications. First, the ligands in the coordination sphere of the incoming metal ions determine the kinetics of the reaction with Pheo*a* because they limit the concentration of the reactive species. Second, the ligands may also determine the direction of the reaction by tuning the redox potential of the cations. Therefore, with some metal ions, when redox reactions may be involved, it is not always of benefit to use their unprotected forms in metalation reactions. The best example is acetate, which on the one hand limits the concentration of reactive M^2+^ species but on the other lowers the M^2+^ oxidative potential. An analogous principle applies to solvents. Thus, in spite of the exceptionally high rate of metalations in MeNO_2_, the synthetic application of this solvent is limited because it does not provide adequate protection against redox-active metal ions and the solubility of many inorganic salts in it is poor.

## Electronic supplementary material

Below is the link to the electronic supplementary material.

Supplementary material 1 (PDF 275 kb)
