# Supplementary material for: Factors controlling the reactivity of divalent metal ions towards pheophytin a
Source: J Biol Inorg Chem. 2017 Jun 21;22(6):941–52. doi: 10.1007/s00775-017-1472-1 (PMC5517585; doi:10.1007/s00775-017-1472-1)
Supplement: Supplementary file 1 — Supplementary material 1 (PDF 275 kb) [file 775_2017_1472_MOESM1_ESM.pdf]

Factors controlling the reactivity of divalent metal ions towards pheophytin *a*

Orzeł et al.

ELECTRONIC SUPPLEMENTARY MATERIAL

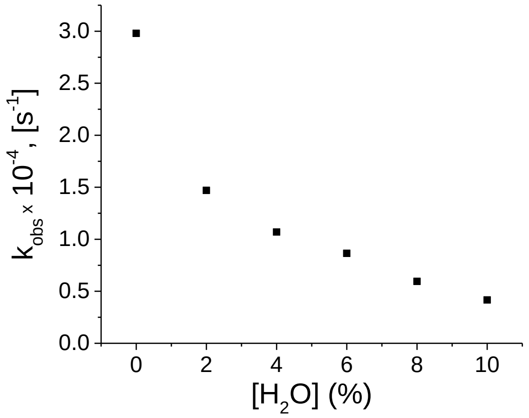

Figure S1. Dependence of the observed rate constant on the water content for pheophytin *a* metalation with zinc trifluoromethanesulfonate in methanol.

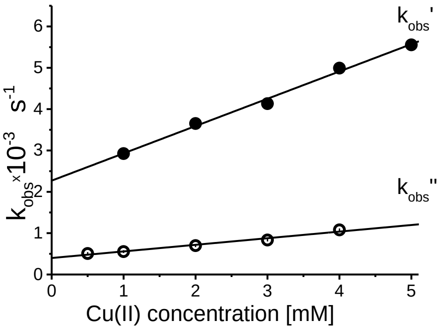

Figure S2. Dependence of the observed rate constant of pheophytin *a* metalation with Cu(II) perchlorate in methanol. Kinetic traces for the reaction reveal biexponential course, hence two reaction rate constants, k_{obs}' and k_{obs}'' (see the text for details).

TABLES

Table S1. Kinetic parameters of the reactions of pheophytin *a* with various divalent metal ions in the presence of weakly coordinative counter ions ($CF_3SO_3^-$, ClO_4^- or NO_3^-) in organic solvents, determined from the decay of the Q_Y band. The values of τ and k_{obs} were determined for $c_{M(II)}$ = 5 mM at 298 K (see the text for details).

Parameter	Solvents	Cd^{2+}	Co^{2+}	Cu^{2+}	Ni^{2+}	Pb^{2+}	Zn^{2+}
τ	MeOH	6.5 min	2.5 h	3.7 min	∞	20 min	2 h [a]
	ACN	∞	20 h	ra	∞	Ls	60 s [a]
	DMF	9.1 h	∞	ra	∞	3.5 h	7.6 h
k_{obs} [s^{-1}]	MeOH	1.77×10^{-3}	3.59×10^{-4}	5.56×10^{-3}	nd	6.20×10^{-4}	1.14×10^{-4} [a]
	ACN	nd	9.10×10^{-6}	ra	nd	Ls	1.04×10^{-2} [a]
	DMF	2.02×10^{-5}	nd	ra	nd	6.42×10^{-5}	1.51×10^{-5}
k_1 [$s^{-1}M^{-1}$]	MeOH	$2.7 \pm 0.6\times10^{-1}$	$7.2 \pm 0.8\times10^{-2}$	$6.6 \pm 0.3\times10^{-1}$	nd	$1.8 \pm 0.4\times10^{-1}$	$8.8 \pm 1.3\times10^{-3}$ [a]
	ACN	nd	$3.9 \pm 0.4\times10^{-4}$	ra	nd	Ls	$2.6 \pm 0.1\times10^{0}$ [a]
	DMF	$7.0 \pm 0.2\times10^{-3}$	nd	ra	nd	$1.4 \pm 0.1\times10^{-2}$	$1.3 \pm 0.1\times10^{-6}$
k_2 [s^{-1}]	MeOH	$2.0 \pm 0.1\times10^{-3}$	$8.2 \pm 0.2\times10^{-6}$	$2.3 \pm 0.1\times10^{-3}$	nd	$1.1 \pm 1.0\times10^{-4}$	$7.1 \pm 0.4\times10^{-5}$ [a]
	ACN	nd	$3.6 \pm 0.1\times10^{-6}$	ra	nd	ls	$3.8 \pm 0.3\times10^{-1}$ [a,c]
	DMF	$1.9 \pm 0.5\times10^{-6}$	nd	ra	nd	$5.3 \pm 0.5\times10^{-6}$	$8.9 \pm 0.4\times10^{-6}$

ra – redox active, nd – not determined, ls – low solubility, ∞ - very slow reaction and high contribution from degradation processes, [a] taken from [1], [c] in [$M^{-1}s^{-1}$]

Table S2. Kinetic parameters of the reactions of pheophytin *a* with chlorides of various divalent metal ions in organic solvents, determined from the decay of the Q_Y band. The values of τ and k_{obs} were determined for $c_{M(II)}$ = 5 mM at 298 K (see the text for details).

Parameter	Solvents	Co^{2+}	Cu^{2+}	Mn^{2+}	Sn^{2+}	Hg^{2+}	Zn^{2+}
τ	MeOH	∞	1.7 min	∞	12 min	4 min	7.3 min
	ACN	1 h	ra	ls	0.5 s	15.5 min	1.5 min
	DMF	∞	1.1 h	8 h	5.5 min	Ls	∞
k_{obs} [s^{-1}]	MeOH	nd	1.74×10^{-2}	nd	4.03×10^{-4}	4.75×10^{-3}	2.04×10^{-3}
	ACN	1.57×10^{-4}	ra	ls	1.03×10^{0}	7.94×10^{-4}	8.51×10^{-3}
	DMF	nd	2.81×10^{-4}	5.21×10^{-5}	1.9×10^{-3}	Ls	nd
k_1 [$s^{-1}M^{-1}$]	MeOH	nd	$3.2 \pm 0.1\times10^{0}$	nd	$2.9 \pm 0.3\times10^{-1}$	$7.7 \pm 0.7\times10^{-1}$	$3.3 \pm 0.5\times10^{-1}$
	ACN	$3.0 \pm 0.4\times10^{-2}$	ra	ls	$2.9 \pm 0.3\times10^{1}$	$1.3 \pm 0.1\times10^{-1}$	$1.9 \pm 0.2\times10^{0}$
	DMF	nd	$4.7 \pm 0.3\times10^{-2}$	$9.1 \pm 1.0\times10^{-3}$	$2.6 \pm 0.8\times10^{-1}$	Ls	nd
k_2 [s^{-1}]	MeOH	nd	$1.1 \pm 0.1\times10^{-3}$	nd	$5.2 \pm 0.9\times10^{-5}$	$8.9 \pm 6.3\times10^{-4}$	$2.7 \pm 1.6\times10^{-4}$
	ACN	$6.6 \pm 0.9\times10^{-5}$	ra	ls	$8.8 \pm 0.1\times10^{-1}$	$3.5 \pm 2.5\times10^{-5}$	$7.9 \pm 6.3\times10^{-4}$
	DMF	nd	$1.1 \pm 0.9\times10^{-7}$	$5.4 \pm 2.7\times10^{-6}$	$7.6 \pm 2.4\times10^{-4}$	Ls	nd

ra – redox active, nd – not determined, ls – low solubility

Table S3. Kinetic parameters of the reactions of pheophytin *a* with acetates of various divalent metal ions in organic solvents, determined from the decay of the Q_Y band. The values of τ and k_{obs} were determined for $c_{M(II)}$ = 5 mM at 298 K (see the text for details).

Parameter	Solvents	Cd^{2+}	Cu^{2+}	Mn^{2+}	Ni^{2+}	Pb^{2+}	Zn^{2+}
τ^a	MeOH	4.3 min	1.7 min	1h	0.8 h	56 min	1.5 min
	ACN	ls	15 min	ls	ls	Ls	58 min
	DMF	5.4 h	6.1 h	ls	∞	23 min	5.5 h
$k_{obs}{}^a$ [s^{-1}]	MeOH	1.90×10^{-3}	5.95×10^{-3}	3.74×10^{-4}	1.69×10^{-3}	8.49×10^{-4}	7.70×10^{-3}
	ACN	ls	6.89×10^{-4}	ls	ls	Ls	1.94×10^{-4}
	DMF	3.31×10^{-5}	5.71×10^{-5}	ls	nd	2.64×10^{-4}	3.24×10^{-5}
k_1 [$s^{-1}M^{-1}$]	MeOH	$3.3 \pm 0.3\times10^{-1}$	$7.2 \pm 0.5\times10^{-1d}$	$1.2 \pm 0.1\times10^{-1}$	$3.4 \pm 0.4\times10^{-1}$	$1.7 \pm 0.2\times10^{-1}$	$1.3 \pm 0.1\times10^{0}$
	ACN	ls	$5.3 \pm 0.1\times10^{-4d}$	ls	ls	Ls	$4.3 \pm 0.9\times10^{-2}$
	DMF	$4.8 \pm 0.4\times10^{-3}$	$4.4 \pm 0.5\times10^{-3}$	ls	nd	$3.9 \pm 0.4\times10^{-2}$	$7.9 \pm 0.6\times10^{-4}$
k_2 [s^{-1}]	MeOH	$2.3 \pm 1.0\times10^{-4}$	$6.7 \pm 0.8\times10^{-4d}$	$1.7 \pm 0.3\times10^{-4}$	$1.9 \pm 0.9\times10^{-4}$	$4.8 \pm 0.1\times10^{-4}$	$1.4 \pm 0.3\times10^{-3}$
	ACN	ls	$7.0 \pm 2.0\times10^{-5d}$	ls	ls	ls	$2.8 \pm 5.6\times10^{-5}$
	DMF	$6.9 \pm 1.3\times10^{-6}$	$3.5 \pm 0.1\times10^{-5}$	ls	nd	$6.7 \pm 1.1\times10^{-5}$	$1.7 \pm 0.1\times10^{-5}$

ls – low solubility, [d]taken from ref. [35]

Table S4. Dissociation products and changes of dissociation energies (ΔE) accompanying solvent release from $Zn(solvent)_4^{2+}$ complexes according to the DFT calculation (see the text for details).

reaction	ΔE [kcal/mol]
$Zn(MeCN)_4^{2+} \rightarrow Zn(MeCN)_3^{2+}$ + solvent	44.1
$Zn(DMF)_4^{2+} \rightarrow Zn(DMF)_3^{2+}$ + solvent	42.6
$Zn(MeOH)_4^{2+} \rightarrow Zn(MeOH)_3^{2+}$ + solvent	39.3
$Zn(MeCN)_4^{2+} \rightarrow Zn^{2+}$ + 4 MeCN	375
$Zn(DMF)_4^{2+} \rightarrow Zn^{2+}$ + 4 DMF	399
$Zn(MeOH)_4^{2+} \rightarrow Zn^{2+}$ + 4 MeOH	314

Table S5. Charges accumulated on the zinc cation in both four- and three-coordinate complexes according to the Loewdin population analysis within DFT calculation (see the text for details). Additionally, dielectric constants of the studied solvents are listed.

solvent	ε	$q(Zn^{2+})$ in $Zn(solvent)_4^{2+}$	$q(Zn^{2+})$ in $Zn(solvent)_3^{2+}$
MeCN	37.5	0.44	0.65
MeOH	32.7	0.31	0.53
DMF	36.7	0.29	0.48
